# Arena3D: visualizing time-driven phenotypic differences in biological systems

**DOI:** 10.1186/1471-2105-13-45

**Published:** 2012-03-22

**Authors:** Maria Secrier, Georgios A Pavlopoulos, Jan Aerts, Reinhard Schneider

**Affiliations:** 1 Structural and Computational Biology Unit, European Molecular Biology Laboratory (EMBL), Meyerhofstrasse 1, Heidelberg 69117, Germany; 2 Department of Electrical Engineering, ESAT-SCD, Katholieke Universiteit Leuven, Leuven, Belgium; 3 IBBT-K.U. Leuven Future Health Department, Leuven, Belgium; 4 Luxembourg Centre for Systems Biomedicine (LCSB), University of Luxembourg, Campus Belval, 7, avenue des Hauts-Fourneaux, Esch sur Alzette, L-4362 Luxembourg

## Abstract

**Background:**

Elucidating the genotype-phenotype connection is one of the big challenges of modern molecular biology. To fully understand this connection, it is necessary to consider the underlying networks and the time factor. In this context of data deluge and heterogeneous information, visualization plays an essential role in interpreting complex and dynamic topologies. Thus, software that is able to bring the network, phenotypic and temporal information together is needed. Arena3D has been previously introduced as a tool that facilitates link discovery between processes. It uses a layered display to separate different levels of information while emphasizing the connections between them. We present novel developments of the tool for the visualization and analysis of dynamic genotype-phenotype landscapes.

**Results:**

Version 2.0 introduces novel features that allow handling time course data in a phenotypic context. Gene expression levels or other measures can be loaded and visualized at different time points and phenotypic comparison is facilitated through clustering and correlation display or highlighting of impacting changes through time. Similarity scoring allows the identification of global patterns in dynamic heterogeneous data. In this paper we demonstrate the utility of the tool on two distinct biological problems of different scales. First, we analyze a medium scale dataset that looks at perturbation effects of the pluripotency regulator Nanog in murine embryonic stem cells. Dynamic cluster analysis suggests alternative indirect links between Nanog and other proteins in the core stem cell network. Moreover, recurrent correlations from the epigenetic to the translational level are identified. Second, we investigate a large scale dataset consisting of genome-wide knockdown screens for human genes essential in the mitotic process. Here, a potential new role for the gene *lsm14a *in cytokinesis is suggested. We also show how phenotypic patterning allows for extensive comparison and identification of high impact knockdown targets.

**Conclusions:**

We present a new visualization approach for perturbation screens with multiple phenotypic outcomes. The novel functionality implemented in Arena3D enables effective understanding and comparison of temporal patterns within morphological layers, to help with the system-wide analysis of dynamic processes. Arena3D is available free of charge for academics as a downloadable standalone application from: http://arena3d.org/.

## Background

Mapping the phenome in the context of dynamic genetic factors is becoming one of the main interests of biology nowadays. There is an increasing amount of data originating from time-resolved imaging experiments on RNA interference screens, synthetic lethality or other systemic perturbations [1-4]. The storage and analysis of this data is however quickly becoming a daunting task. Given this current inflow of time-resolved data, the necessity of developing tools capable of dealing with large amounts of temporal information is hence becoming increasingly evident.

The phenotypic landscape reflects the robustness of the underlying genetic networks and its understanding should help in elucidating the reverse rewiring of genetic circuits. The dynamic factor in biological systems adds another dimension of complexity and plays a major role in understanding the process. Therefore the common approach of excessively simplifying the dynamic factor will result in a potentially critical loss of understanding. Visualization tools can greatly enhance the ability to perceive this type of complex data.

Arena3D has been previously developed as a visualization and analysis platform for the display and understanding of connections between different data types of biological information [5]. It uses a multi-layered concept to allow the visualization of networks and links between them in three-dimensional space. Each layer represents one type of biological category (genes, proteins, structures, diseases etc.) and the nodes on different layers are connected according to known or predicted relations between them. Different clustering algorithms are available to order the nodes according to similarity measures. Here we report on Arena3D version 2.0 that extends the capabilities of the application by incorporation of time course data handling through animations, clustering, tracking and similarity scoring. It allows the direct visualization of comparative changes and time patterns for different phenotypes, tissues, cellular compartments or other parallel layers of biological information. The upgrade considerably enhances the ability to interpret small to medium-sized datasets of time-resolved information in the context of genotype-phenotype landscape mapping. Furthermore, it introduces a new concept of dynamic 3D data visualization for extensive phenotypic studies.

While different tools for visualizing time course data, gene expression and network clusters already exist, e.g. VistaClara [6], GENeVis [7-9], Pathline [10], GATE [11], clusterMaker [12], Prism [13], Arena3D has several advantages over them. It uses a unique multi-layered concept of displaying networks in 3D, which includes: data integration (using different layers for different data types), time course data (including movie generation), gene expression data (changes of gene expression over time). It can handle both non-time series and time series data and, for the latter, comparison between different networks or phenotypes can be easily performed. Arena3D enables tracking of individual genes, a feature that is not encountered in most of the software mentioned. Thus, it enables focused analysis, as well as global comparisons and classification into categories. Arena3D offers more flexibility in laying out the networks compared to GATE or GENeVis and also the possibility to compare networks over time. Moreover, it does not require a hierarchy and can handle larger amounts of data than Pathline. Besides clustering abilities similar to clusterMaker or gene expression tracking like in VistaClara or Prism, Arena3D enables measurements of overall time series similarity of genes and of networks. However, it does not offer a heat map view and its clustering methods are less diverse compared to clusterMaker. The tool is generic and can be used even for non-biological applications, whereas the other tools mentioned are more specific. The combination of dynamic information visualization, 3D layout and similarity classification make it a useful tool for phenotypic comparison studies in a genetic network contextual background.

## Implementation

Arena3D was implemented using Java (JDK 1.6) and Java3D (1.6.1 API). The JFreeChart library [14] is used for the line plot view of time course values upon node click events. The software is available as a standalone application downloadable from the website. The Java Runtime Environment http://www.java.com/ and Java3D libraries http://java3d.java.net/ are required for running Arena3D on any operating system and Macintosh users should also install the JOGL libraries http://opengl.j3d.org/. Simple API implementation for plug-in development is planned for the future. The source code is available for download for users that wish to customize their analysis.

The nodes are colored according to the associated values of the respective biological entities on a yellow-blue color scale, with grey representing absolute zero (or the cases where there is no value associated to the node). The conversion of the values to the scale is calculated such that the colors map from yellow to blue to the interval *(minValue, maxValue)*, where *minValue *is the absolute minimal value that any node may have throughout the time course for the respective layer and *maxValue *is the absolute maximal one. The gradient colors can be customized by the user. The option of using other colorblind-safe gradients is also offered. The scale is mapped separately for each layer, as there may be cases where the parameters measured for different layers of information are not comparable in magnitude or units of measurement. Caution should therefore be taken when interpreting results from comparisons among different layers based on color alone.

### Statistical calculations

To compute and graphically display correlations between the time-resolved vectors associated to each node (representing a gene/protein or other biological entity) the Pearson correlation calculation has been used. Only correlations with a certain p-value (0.10, 0.05, 0.02 or 0.01) are displayed. By default, correlations with a p-value of 0.05 will be shown. The significance of the correlation is assessed according to the Pearson product-moment correlation coefficient (PMCC) table of critical values, which describes the minimal Pearson correlation coefficient values for a certain level of significance depending on the number of degrees of freedom. Importantly, for this correlation measure the data is assumed to be normally distributed.

As a non-parametric alternative to the Pearson correlation calculation, the Spearman rank correlation is also available for the user (results not shown). This is a better measure for the cases when the data is not normally distributed. The significance of the Spearman correlation r is assessed using the following formula:

(1) $$t=r\sqrt{\frac{n-2}{1-r^{2}}}$$

This has an approximate Student's t distribution with *n*-2 degrees of freedom under the null hypothesis, where *n* is the number of time points in the series [15].

It is important to note that, since the different samples in the time series data are not independent, the current correlation measurements are limited and the results should be interpreted with care. They are meant only to provide a first rough indication of similarity between time series, using very simplified assumptions. Extensions to non-parametric association measures taking into account the dependence between columns [16,17], as well as multiple testing corrections (e.g. Benjamini-Hochberg false discovery rate [18]) are planned for the future.

The option to score genes by similarity of the associated time-resolved vectors relies on two scoring schemes, such that the score for each gene is computed either as: (a) the average of the vector values; or as (b) the lower bound of the Wilson score confidence interval for a Bernoulli parameter as in:

(2) $$S\left({\text{g}}_{i},\alpha \right)=\frac{\hat{\text{p}}+\frac{z_{\alpha /2}^{2}}{2n}\pm z_{\alpha /2}\sqrt{\frac{\hat{\text{p}}\left(1-\hat{\text{p}}\right)+\frac{z_{\alpha /2}^{2}}{4n}}{n}}}{1+\frac{z_{\alpha /2}^{2}}{n}},t\in \left\{0..N\right\}$$

for every gene g_i_, *i ∈ *{1..*M*} (*M *being the total number of genes), where *p *represents the fraction of positive ratings, *z*_*α*/2 _is the (1-*α*/2) quantile of the Gaussian distribution and *n *is the number of ratings [19,20]. The latter scoring should balance the proportion of positive ratings with the uncertainty of a small number of observations.

The scores are then converted to a scale from 0 to 10 and assigned to bins correspondingly, such that the colors of the bins reflect the magnitude of the score and genes with similar scoring are colored identically. A color scale from white to red is used for this purpose, as depicted in the following section.

### Clustering of values for individual time points

The clustering of genes at individual time points is performed separately for every layer based on distance geometry of the values associated to the genes for the respective layer. Given a distance matrix between a set of points, the distance geometry algorithm calculates the coordinates of each point in 3D space, and subsequently places the nodes with shortest scoring distance closer to each other, as described in [21]. For this algorithm one does not need to specify the number of clusters the genes should be classified into, but rather places them in close proximity according to the distance matrix. The clustering is performed purely for visualization purposes, for faster identification of genes with similar phenotypic time course profiles, and does not affect any of the results. The user can employ a different clustering algorithm of his wish at any point during the analysis.

## Results and discussion

**Figure 1:**
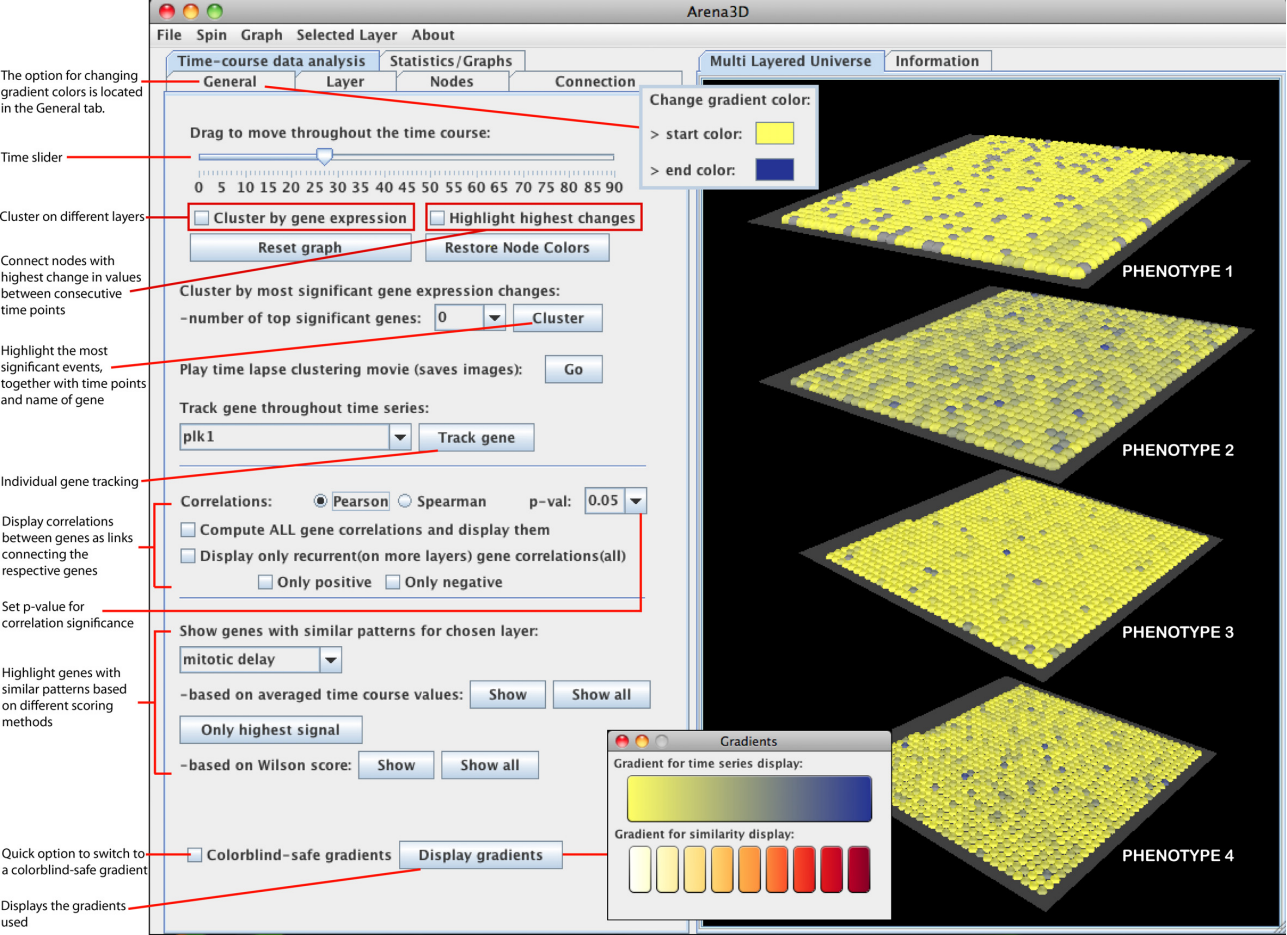
**Panel with time-course data analysis features**. The main functionality implemented for dealing with time-resolved data and how it can be accessed is pinpointed.

Several new features have been implemented in version 2.0. The main enhancements deal with analyzing time-course multiple-level data through: (a) changes in gene expression, protein concentration or other parameters tracked through color changes; (b) clustering of entities on different layers based on associated values; (c) individual gene tracking; (d) display of gene correlations; (e) scoring and coloring based on similarity features. The color scheme used for tracking time-resolved changes or for similarity depiction can be changed according to the user's wish, including gradients visible for colorblind people. A more detailed listing of the new features can be found in Table 1. These can be accessed within the application as described in Figure 1.

**Table 1:** Arena3D versus previous version

Functionality	Previous versions	Arena3D 2.0
Load network data	x	x

Load time course data		x

Layouts		
*Circular*	x	x
*Grid*	x	x
*Spherical*	x	x
*Hierarchical*	x	x

Clustering		
*Fruchterman - Reingold*	x	x
*Distance Geometry*	x	x
*Affinity Propagation*	x	x
*Markov Clustering*	x	x
*K-Means*	x	x
*Neighbor Joining*	x	x
*UPGMA*	x	x

Move nodes	x	x

Move/scale/spin layers	x	x

Time course data analysis		
*Time slider*		x
*Cluster by gene expression*		x
*Highlight peaks*		x
*Cluster by top expression changes*		x
*Play animation*		x
*Individual gene tracking*		x
*Pearson/Spearman correlation display*		x
*Similarity scoring*		x
*Choose color scheme*		x
*Colorblind-safe color scheme*		x

Network export		
*Medusa format*	x	x
*Pajek format*	x	x
*VRML format*	x	x
*JPEG format*	x	x

Listed are the different functionality improvements with respect to the previous version of the software.

We illustrate these features by application to two datasets from time-resolved genotype-phenotype experiments. The corresponding files in Arena3D format for the two case studies are available as Additional file 1 archive.

### Experimental case study 1: system-level differences in the epigenetic, transcriptional and translational dynamics of embryonic stem cells

Phenotypic differences arising from the downregulation of potent regulatory factors in the cell propagate at various levels, from epigenetic to organismal. An illustrative example is the one that has been recently studied for the downregulation of the pluripotency regulator Nanog. The results synthesize a systems-level analysis of dynamic changes in embryonic stem cells (ESCs) upon downregulation on three different layers: epigenetic, transcriptional and translational. The dataset contains measurements of histone acetylation, RNA polymerase II localization, mRNA abundance and protein levels for a set of genes [22]. We look at the dynamic changes within the core ESC protein-protein interaction network, as defined in [22] (see Additional files 2 and 3 for the description of the genes used to perform this analysis and their time course values). The changes are recorded for three time points (days 1, 3 and 5). We show how Arena3D functionality enables us to find interesting patterns in the data not identified in the original paper, like patterns of perturbation propagation from the epigenetic to the translational level or recurrent correlations in dynamic changes throughout all systemic layers.

**Figure 2:**
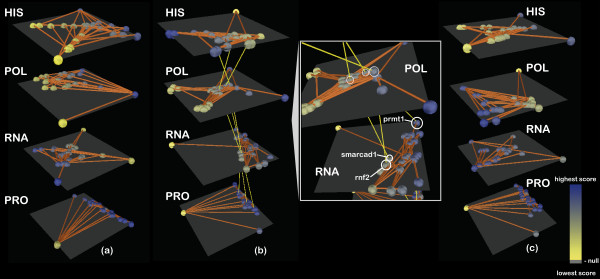
**Dynamic clustering of layered biological profiles**. The network of ESC core genes is shown connected on each one of the four layers depicting phenotypic outcomes in terms of histone acetylation levels (HIS), RNA polymerase binding affinity (POL), mRNA production (RNA) and protein translation levels (PRO) upon knockdown of *nanog*. Nodes correspond to genes and are colored according to the values associated at every time point for each informational level, on a yellow-to-blue gradient as indicated. Clustering at three distinct time points is shown for each level: (**a**) day 1; (**b**) day 3; (**c**) day 5. There seems to be a transition in terms of dynamics based on the evolution of gene-associated values and clustering outcomes from the epigenetic levels (most dynamic) to the translational level (most stable). Genes with highest change in associated impact value between consecutive time points are connected between layers for better emphasis (b): close-up picture shows genes *prmt1*, *smarcad1 *and *rnf2 *as the ones displaying the highest change.

The four layers of systems dynamics are visualized correspondingly: histone acetylation, chromatin bound RNA polymerase II, mRNA levels and nuclear protein abundance. On each layer, the ESC core network is represented, with nodes corresponding to genes/proteins and links to the interactions between them. Nodes are colored according to the level of acetylation, polymerase localization on chromatin, mRNA abundance or protein levels, respectively, for the corresponding gene. Values map to node color on a yellow-blue color scale, such that lowest values are coded in blue, highest in yellow and the intermediate ones according to the gradient in-between. Grey represents absolute 0. The changes in these values for the three days of measurement can be easily tracked using a slider that updates the network and the node colors for every time point. One can then further analyze snapshots of phenotypic profiles for different stages of the experiment (Figure 2).

#### Dynamic clustering on different layers

In order to get a feeling of how similar the biological entities are on each layer, clustering is enabled for individual layers separately: in this way, one can compare different phenotypic layers in terms of how the genes cluster according to their impact values. To enable this, the user must select "Cluster by gene expression" in the "Time-course data analysis" tab in the application and then move through time using the slider. Furthermore, gene-associated values change throughout the time course, but sudden peaks or declines often prove particularly more interesting than individual values at time points. We enable fast discovery of genes exhibiting this behavior by highlighting those that have the highest change in impact between two consecutive time points. Such a gene will be connected throughout all layers for easy recognition. To enable this feature, "Highlight highest changes" must be selected. The clustering changes dynamically at each time point.

Clustering on different levels for different time points as shown in Figure 2 reveals that downregulation of *nanog *strongly reflects in dynamic changes at the epigenetic level, but less prominent at the transcriptional (mRNA) and translational (protein) level. The genes/proteins seem to maintain similar levels of abundance and similar clustering in time for the last two levels, which indicates a dampening of the perturbation induced at the chromatin level. The highest impact changes are noticed for genes *smarcad1 *(SWI/SNF-related, matrix-associated actin-dependent regulator of chromatin [Ensembl:ENSG00000163104]), *prmt1 *(an arginine methyltransferase [Ensembl:ENSG00000126457]) and *rnf2 *(ring finger protein belonging to the Polycomb group [Ensembl:ENSG00000121481]), which are highlighted by connecting throughout the layers (Figure 2b). Given that all these proteins act at the level before mRNA production, the intensity of signal being higher for epigenetic levels is justified. The strong impact change of all three genes upon *nanog *downregulation is rather puzzling, as *smarcad1, prmt1 *and *rnf2 *only interact with *nanog *through mediators *rex1 *and *nac1 *(whose values do not change throughout the experiment) and are situated at the periphery of the ESC network [22]. This could suggest that there may be an alternative route from *nanog *to the respective genes that makes them so susceptible to the impact of downregulation.

#### Correlation display

Gene pairs with a significant positive or negative correlation in expression can be identified by connecting the two genes with a line colored correspondingly. We exemplify this by looking at the Pearson correlations, but the same workflow can be applied when the user wants to use the Spearman rank correlations instead. First, the correlation algorithm (Pearson/Spearman) should be chosen. In the case of the Pearson correlation coefficient, significance of correlation is assessed according to the PMCC table of critical values, as described in the previous section. Visualizing this type of relationship for different layers allows us to identify recurrent correlations between pairs of genes for different biological measurements, from chromatin modifications to protein abundance.

To display all significant correlations, the user must select "Compute ALL gene correlations and display them" in the "Correlations" section. For recurrent correlations only, the "Display only recurrent (on more layers) gene correlations" will be selected, and the options of only displaying positive or only negative correlations are available. The p-value can be set to a desired threshold.

**Figure 3:**
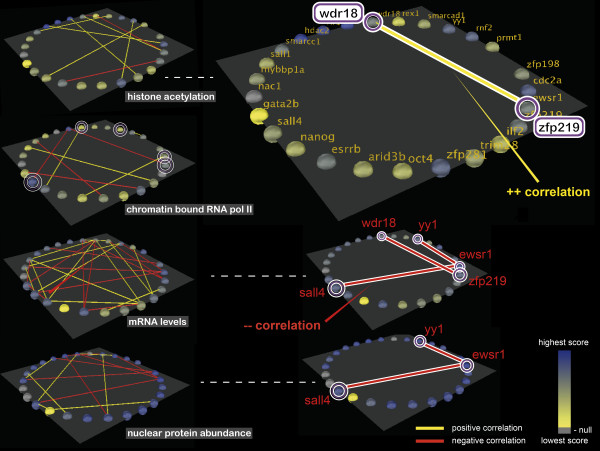
**Recurrent correlations display from the epigenetic to the protein level**. Phenotypic outcomes at the level of histone acetylation, chromatin binding, mRNA production and nuclear protein abundance are shown for the genes that form the ESC core network on each layer. Nodes are colored corresponding to the gene value on a yellow-blue color scale. Correlations between the vectors of time-resolved values associated to each gene are highlighted by connecting the corresponding nodes with a yellow line (for positive correlations) or a red line (for negative correlations). Left hand side: all correlations with p-value < 0.05 (i.e. coefficient greater than 0.997) are represented as connections between nodes for each layer. Right hand side: only recurrent correlations on at least two layers are displayed for the corresponding layers. The layer of chromatin bound RNA polymerase II is not shown because there are no recurrent correlations on that layer.

Even though for the given data there are only three time points (degree of freedom equal to 1), which could be considered insufficient for significant correlations, we do find several cases when the correlation coefficient is greater than 0.997, such that the p-value is less than 0.05, thus denoting significant correlations (Figure 3, left hand side). For illustration purposes we consider this sufficient. However, the assessment of whether the data volume is suitable for applying such calculations should be done on a case-by-case basis.

The right hand side of Figure 3 shows the different patterns of recurrent correlations at systemic level. Gene pairs *yy1 *- *ewsr1 *and *sall4 *- *ewsr1 *are negatively correlated at the level of mRNA production, as well as the protein level. This means that there are post-transcription factors that make the two genes differ in the mRNA expression level, perhaps within the processing of mRNA precursors, which will consequently lead to negatively correlated levels of protein obtained. Even more interesting patterns are obtained for genes *wdr18 *and *zfp219*. They are positively correlated in the proportion of histone acetylation, but negatively correlated in terms of mRNA levels and uncorrelated for the other levels. This indicates subsequent steps after acetylation that lead to differentiation of mRNA and protein production levels.

While these examples show that there is a high level of heterogeneity from the epigenetic down to the translational level, we can also observe that a couple of correlations are rather uniform throughout layers. Similar or recurring patterns are particularly noticeable between the mRNA and protein level, which is in concordance with the fact that protein and mRNA copy numbers correlate, despite the fact that their half-lives do not [23].

### Experimental case study 2: profiling phenotypic defects in cell division upon single perturbations in the system

Gene knockdown studies have been performed extensively in high-throughput experiments and the outcome is often challenging to analyze. One of the interesting examples that has come up lately in the literature looks at cell division defects derived from suppression of genes essential to the cell cycle. This large scale experiment was performed on HeLa cells and consists of siRNA knockdown screens for genes involved in cell division, as described in [24]. The knockdown outcomes are followed through time-lapse imaging of the cells and the observed cell division defects are classified into seven main phenotypes: mitotic delay, binuclear, polylobed, grape, large, dynamic and apoptosis. For each gene that upon knockdown causes problems in cell division a vector of time-point values is assigned, summarizing the penetrance of each phenotype in the cell population at each time point through a score based on morphological features. We look at a total number of 90 time points, spanning 45 hours of cell life.

**Figure 4:**
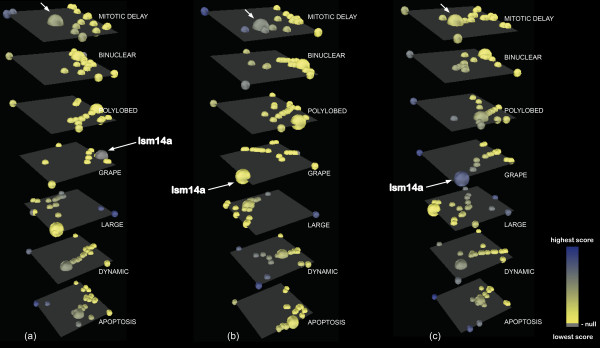
**Time-resolved clustering and individual tracking of a gene**. A subset of essential mitotic genes (see Additional file 4) is depicted on each layer as nodes colored according to the associated knockdown effect, from yellow to blue (low to high impact). Grey represents 0 impact. Each layer corresponds to one phenotype. Clustering of gene knockdown profiles and gene tracking are highlighted for three individual time points: (**a**) t = 2 h; (**b**) t = 7 h; (**c**) t = 33 h. Dynamic clustering of genes on different layers reveals more dynamic changes for the "grape", "large" and "dynamic" phenotypes compared to "mitotic delay" or "polylobed", which tend to stay more constant, indicating that these phenotypes may be more stable compared to the previous ones. The gene *lsm14a *is tracked by node expansion (also indicated using arrows for "mitotic delay" and "grape"). Its silencing has a mild to more pronounced impact for the "mitotic delay" phenotype (a-b), while having no influence on phenotype "grape" in the beginning (a) and high towards the end (c), indicating a latent impact on the cell upon this particular knockdown that determines it to adopt "grape" morphology after stagnation during mitosis.

We visualize the effects of every gene knockdown (represented by nodes) for every resulting phenotype (each represented in one separate layer). The dynamic changes in gene knockdown impact are visualized through corresponding changes in node color as described for the previous experiment. The changes can be again tracked, as shown in Figure 4. The same visualization can also be applied to other datasets for changes in gene expression, protein concentration or any other kind of time-resolved variables.

#### Clustering knockdown outcomes

Dynamic clustering performed for a selected subset of genes from this dataset as chosen in [24] (see Additional files 4 and 5 for details) reveals comparative patterns of more resistant or more volatile phenotypes: Figure 4 shows how phenotypes "mitotic delay", "binuclear" or "polylobed" tend to preserve similar clustering patterns throughout time. In contrast, the other morphological categories display more frequent changes, indicating that they are intermediate phenotypes rapidly succeeded by others within the cell population. The "apoptosis" phenotype is revealed to be rather dynamic, which might seem counterintuitive at first, but in fact is not: the effects are measured at the level of cell populations and not individual cells, so in one plate there will be a constant turnover of cells that divide with/without defects and then die, followed by other cells that start dividing and so on - hence making apoptosis not a permanent but rather a cyclic phenotype. Clustering enables positioning a certain gene of interest and observing how its impact compares to other genes whose suppression results in a particular phenotype, as explained in the next subsection.

#### Individual gene tracking

If one is interested in following the patterns in time for a particular gene, tracking of the corresponding node is enabled through an increment in node size. In this way, one can easily observe how the gene's knockdown effect changes through time, how it clusters with effects of other genes and how similar its behavior is to others. To enable gene tracking, the user must select the gene of interest from the section "Track gene throughout time series" in the application and then click the corresponding button for tracking.

Figure 4 shows how the gene *lsm14a *that is being tracked reveals a latent effect upon knockdown of determining the cells to slowly assume the grape morphology, which, remarkably, is a "rare" phenotype. Grape is termed a "rare" phenotype because there are very few cells that adopt this morphology upon perturbation, which makes it interesting to study for understanding the causative factors. Additionally, comparative tracking of gene *lsm14a *on the different phenotypic layers at different time points enables identification of ordered phenotypic succession processes: the cells seem to exhibit a transition from mitotic delay to grape, as *lsm14a *shows a mild "mitotic delay" phenotype in Figure 4a) and more pronounced in 4b), after which the phenotype "grape" becomes prominent in 4c).

*lsm14a *is an Sm-like protein believed to be involved in pre-mRNA splicing and the formation of P-bodies [25-27]. There is also evidence that it becomes associated with the mitotic spindle [28], suggesting that its knockdown might cause problems in spindle assembly. This analysis enables us to obtain further hypotheses about potential functions of the gene *lsm14a*. Considering the effects of its knockdown, there are indications that it could be involved not only in karyokinesis, but also in cytokinesis processes, as the "grape" morphology exhibits many micronuclei which can be a result of both improper nuclear and cytoplasmic division. This raises interesting observations about the versatility and adaptability of this gene. Further experimental evidence is needed in order to identify the subprocesses in which the product of this gene is involved.

#### Similarity scoring

Given a large set of genes, one would like to find those that have similar patterns through time. This is done by coloring each node according to a similarity score that takes into account the entire vector of values associated to each gene. This further enables straightforward comparison of temporal progression among different layers of information.

The two scoring schemes used allow for rather different interpretations. This is why both options are available to the user to choose the most suitable one to the respective data. To enable coloring based on similarity, the user must select a layer in the section "Show genes with similar patterns for chosen layer" and then click "Show" or "Show all" (for all layers). The corresponding button will be clicked depending on whether one opts for the average or the Wilson scoring scheme.

**Figure 5:**
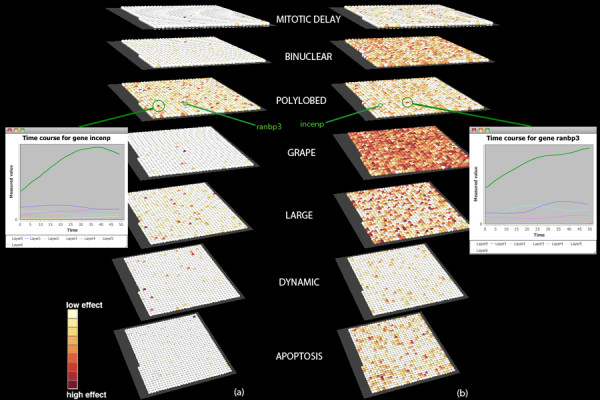
**Similarity scoring of gene knockdown impact profiles**. Scoring the overall impact of individual gene knockdowns on the prevalence of different phenotypes. We look at the span of one cell cycle, approximately 50 time points. Nodes correspond to gene knockdown events and are colored according to the scoring scale, as indicated (white-dark red, low-high). A set of 1067 essential mitotic genes is represented on each layer. One gene has the same position on all layers. Two alternative scoring schemes are presented: (**a**) averaging the values in the gene knockdown vector; (**b**) the lower bound of Wilson score confidence interval. A line chart of timeline evolution of knockdown values for each phenotype can be obtained by clicking on a particular node of interest, as shown for genes *incenp *and *ranbp3*, both of which display increasingly higher signal for the phenotype "polylobed" (green line) throughout the time course.

The score based on averaging (Figure 5a) is revealing some genes with high effect upon knockdown on the cell phenotypic landscape. The highest peaking signals overall are found for the polylobed phenotype, which is indeed a strongly prevalent phenotype in many of the screens. This scoring scheme thus allows selective decisions about potentially interesting targets for further experiments.

On the other hand, the Wilson scoring scheme allows for a more detailed analysis of the true signal within a single phenotype by noise elimination. Figure 5b) reveals several genes scoring high for several morphologies. The intensity of the signal is, however, uniquely scored for every phenotype, such that one cannot compare or make any hypotheses about the "most resistant/susceptible" phenotypes. Caution should be taken when using the Wilson scoring scheme, as the normalization used tends to bring out many points of high signal in a pool where most values are low (e.g., the "grape" phenotype is a rare one but most genes appear to be scoring highly for it because of the normalization effect). To recapitulate, using the latter scoring scheme one can look for true signals within a particular phenotype but not compare among phenotypes.

As highlighted in Figure 5, downregulation of gene *incenp *(an inner centromere protein antigen [Ensembl:ENSG00000149503]) is scored as highly influential for the polylobed phenotype according to scoring scheme (a) and not as much for the same phenotype according to scoring scheme (b). On the other hand, the suppression of gene *ranbp3 *(a RAN binding protein [Ensembl:ENSG00000031823]) receives a high score for the polylobed morphology under the latter scoring scheme and a lower score for the former scoring scheme. The timeline of variation for the two genes is obtained by clicking on the respective nodes and reveals the line chart for the respective genes for all phenotypic layers. Here one can see that in fact both genes have a high signal for the polylobed phenotype. Since gene *ranbp3 *has a lower average than *incenp*, it did not score high by the averaging scheme, but its signal is captured by the second scheme which manages to balance out some of the noise. This shows that similarity scoring performs well in identifying global patterns in the data, especially in the context of a high number of samples, and the two scoring schemes are best used complementarily.

## Conclusions

Genetic pleiotropy and locus heterogeneity are two phenomena that contribute to making the landscape of genotype-phenotype relations progressively intricate [29]. Visualization tools like Arena3D can become a great asset in the attempt to elucidate these connections, especially in a dynamic context.

We have shown how this tool can be used in phenotypic profile classification, as well as in multigene trait prediction from the genotype. The functionality of Arena3D can provide the basis to identifying both rare and prevalent phenotypes and their underlying signalling networks, components of which may be used as markers for diseases.

One of the main assets of this tool is the interactive analysis of temporal data: it enables the discovery of global patterns, but also of time patterns for individual genes of interest, given small to medium datasets with a few or many time points. The advantage is that one can also focus on a particular time point that may stand out as exhibiting interesting behavior of genes/proteins and look deeper into the reasons for this highlight. This approach thus allows for a better understanding of the role time plays within the biological process.

It is becoming increasingly important that the analysis of networks and pathways should switch from a global to a time-resolved, tissue-specific view, as there are essential differences encountered at this level [30]. Analyzing mutational effects by taking into account tissue and organ specificity can provide an insight into developmental patterns of the system. It can also help uncover functional redundancies or complementarities that could be useful for rescuing detrimental phenotypes [29]. In this respect we believe Arena3D will prove particularly suitable, with the ability to compare and contrast expression levels in different tissues over time, opening the path towards a better understanding of cell and tissue-specific regulation and eventually towards differential treatment of diseases.

## Availability and requirements

**Project name**: Arena3D

**Project home page**: http://arena3d.org/

**Operating system(s)**: Platform independent

**Programming language**: Java, Java3D

**Other requirements**: Java 1.6 (or higher)

**License**: Arena3D is available free of charge for academic use.

**Any restrictions to use by non-academics**: Commercial users should contact the authors.

Competing interests

The authors declare that they have no competing interests.

## Authors' contributions

MS implemented the time course data analysis functionality for Arena3D and applied it to biological data. GAP was the first developer of the software and he contributed to the design and the implementation of the software. JA helped in analyzing and addressing specific visual concepts implemented by MS and GAP. RS conceived the concept and the main idea of Arena3D. He designed, coordinated and supervised the project. All authors drafted, read and approved the final manuscript.

## Supplementary Material

Additional file 1 **Files for testing in Arena3D**. Input files in the Arena3D format, for users wishing to test the examples discussed in the paper directly with the software. The archive contains 3 files: ESC_core_network_timeseries_forARENA3D.txt - data for case study 1; mitotic_genes_all_timeseries_forARENA3D.txt - data for case study 2, all genes, 50 time points; mitotic_genes_subset_timeseries_forARENA3D.txt - data for case study 3, defined subset of genes, 90 time points. Click here for file

Additional file 2 **The ESC core network**. List of the genes involved in the ESC core network along with their description from Ensemble release 63 [31], as described in [22]. Click here for file

Additional file 3 **Time series values for genes in the ESC core network**. List of the genes involved in the ESC core network along with their associated time series values for the four levels: histone acetylation, chromatin binding, mRNA and protein levels for the 3 days of the experiment. Click here for file

Additional file 4 **List of potentially interesting mitotic genes**. Subset of genes involved in cell division, chosen according to the targets discussed in [24], along with their description from Ensemble release 63 [31]. Click here for file

Additional file 5 **Time series values for potentially interesting mitotic genes**. Subset of genes involved in cell division, chosen according to the targets discussed in [24], along with their associated time series values corresponding to the seven phenotypes for 90 time points. Click here for file

## References

[B1] Henderson M Gonzales I Arora S Choudhary A Trent J Von Hoff D Mousses S Azorsa D High-throughput RNAi screening identifies a role for TNK1 in growth and survival of pancreatic cancer cells Mol Cancer Res 2011 9 6 724 32 10.1158/1541-7786.MCR-10-0436 21536687 PMC3137903

[B2] Bayona-Bafaluy M Sánchez-Cabo F Fernández-Silva P Pérez-Martos A Enríquez J A genome-wide shRNA screen for new OxPhos related genes Mitochondrion 2011 11 3 467 75 10.1016/j.mito.2011.01.007 21292037

[B3] Cerone M Burgess D Naceur-Lombardelli C Lord C Ashworth A High-throughput RNAi screening reveals novel regulators of telomerase Cancer Res 2011 71 9 3328 40 10.1158/0008-5472.CAN-10-2734 21531765

[B4] Costanzo M Baryshnikova A Bellay J The genetic landscape of a cell Science 2010 327 5964 425 31 10.1126/science.1180823 20093466 PMC5600254

[B5] Pavlopoulos G O' Donoghue S Satagopam V Soldatos T Pafilis ERS Arena3D: visualization of biological networks in 3D BMC Syst Biol 2008 2 104 10.1186/1752-0509-2-104 19040715 PMC2637860

[B6] Kincaid R Kuchinsky A Creech M VistaClara: an expression browser plug-in for Cytoscape Bioinformatics 2008 24 18 2112 4 10.1093/bioinformatics/btn368 18678589 PMC2530886

[B7] Westenberg MA van Hijum SAFT Kuipers OP Roerdink JBTM Visualizing Genome Expression and Regulatory Network Dynamics in Genomic and Metabolic Context Computer Graphics Forum 2008 27 3 887 94 10.1111/j.1467-8659.2008.01221.x

[B8] Westenberg MA van Hijum SAFT Lulko AT Kuipers OP Roerdink JBTM Interactive visualization of gene regulatory networks with associated gene expression time series data Visualization in Medicine and Life Sciences 2007 Berlin: Springer Verlag 293 312

[B9] Bourqui R Westenberg MA Visualizing Temporal Dynamics at the Genomic and Metabolic Level13th Int Conf Information Visualization 2009 317 322

[B10] Meyer M Wong B Styczynski M Pfister H Pathline: a tool for comparative functional genomics Computer Graphics Forum (Proc EuroVis) 2010 29 3 1043 52 10.1111/j.1467-8659.2009.01710.x

[B11] MacArthur BD Lachmann A Lemischka IR Ma'ayan A GATE Software for the analysis and visualization of high-dimensional time series expression data Bioinformatics 2010 26 1 143 4 10.1093/bioinformatics/btp628 19892805 PMC2796822

[B12] Morris JH Apeltsin L Newman AM Baumbach J Wittkop T Su G Bader GD Ferrin TE clusterMaker: a multi-algorithm clustering plugin for Cytoscape BMC Bioinformatics 2011 12 1 436 10.1186/1471-2105-12-436 22070249 PMC3262844

[B13] Wu W Noble WS Genomic data visualization on the Web Bioinformatics 2004 20 11 1804 5 10.1093/bioinformatics/bth154 14988106

[B14] Gilbert D Morgner T JFreeChart, a free Java class library for generating charts http://www.jfree.org/jfreechart

[B15] Kendall MG Stuart A The Advanced Theory of Statistics, Volume 2: Inference and Relationship Griffin 1973 31 19 21

[B16] Kruglyak S Tang H A New Estimator of Significance of Correlation in Time Series Data Journal of Computational Biology 2001 8 463 70 10.1089/106652701753216486 11694177

[B17] Masry E The estimation of the correlation coefficient of bivariate data under dependence: Convergence analysis Statistics & Probability Letters 2011 81 1039 45 10.1016/j.spl.2011.02.026

[B18] Benjamini Y Hochberg Y Controlling the false discovery rate: a practical and powerful approach to multiple testing Journal of the Royal Statistical Society, Series B (Methodological) 1995 57 1 289 300

[B19] Agresti A Coull B Approximate is better than'exact' for interval estimation of binomial proportions The American Statistician 1998 52 119 126 10.2307/2685469

[B20] Wilson E Probable inference, the law of succession, and statistical inference J Am Stat Assoc 1927 22 209 212 10.2307/2276774

[B21] Crippen GM Havel TF Distance Geometry and Molecular Conformation 1988 New York: Wiley

[B22] Lu R Markowetz F Unwin R Leek J Airoldi E MacArthur B Lachmann A Rozov R Ma'ayan A Boyer L Troyanskaya O Whetton A Lemischka I Systems-level dynamic analyses of fate change in murine embryonic stem cells Nature 2009 462 7271 358 10.1038/nature08575 PMC3199216 19924215

[B23] Schwanhäusser B Busse D Li N Dittmar G Schuchhardt J Wolf J Chen W Selbach M Global quantification of mammalian gene expression control Nature 2011 473 7347 337 42 10.1038/nature10098 21593866

[B24] Neumann B Walter T Heriche JK Bulkescher J Erfle H Conrad C Rogers P Poser I Held M Liebel U Cetin C Sieckmann F Pau G Kabbe R Wuensche A Satagopam V Schmitz MHA Chapuis C Gerlich DW Schneider R Eils R Huber W Peters JM Hyman AA Durbin R Pepperkok R Ellenberg J Phenotypic profiling of the human genome by time-lapse microscopy reveals cell division genes Nature 2010 464 721 727 10.1038/nature08869 20360735 PMC3108885

[B25] Marnef A Sommerville J Ladomery M RAP55: insights into an evolutionarily conserved protein family Int J Biochem Cell Biol 2009 41 5 977 81 10.1016/j.biocel.2008.06.015 18723115

[B26] Tanaka K Ogawa K Takagi M Imamoto N Matsumoto K Tsujimoto M RAP55, a cytoplasmic mRNP component, represses translation in Xenopus oocytes J Biol Chem 2006 281 40096 40106 10.1074/jbc.M609059200 17074753

[B27] Yang W Yu J Gulick T Bloch K Bloch D RNA-associated protein 55 (RAP55) localizes to mRNA processing bodies and stress granules RNA 2006 12 547 554 10.1261/rna.2302706 16484376 PMC1421083

[B28] Gache V Waridel P Winter C Juhem A Schroeder M Shevchenko A Popov A Xenopus meiotic microtubule-associated interactome PLoS One 2010 5 2 e9248 10.1371/journal.pone.0009248 20174651 PMC2822853

[B29] Torkamani APL Hegele R Schork N Hegele R Kinase mutations in human disease: interpreting genotype-phenotype relationships Nat Rev Genet 2010 11 1 60 74 10.1038/nrg2707 20019687

[B30] Lopes TJ Schaefer M Shoemaker J Matsuoka Y Fontaine JF Neumann G Andrade-Navarro MA Kawaoka Y Kitano H Tissue-specific subnetworks and characteristics of publicly available human protein interaction databases Bioinformatics 2011 27 17 2414 2421 10.1093/bioinformatics/btr414 21798963

[B31] Flicek P Aken B Ballester B Ensembl's 10th year Nucleic Acids Res 2010 38 D557 D562 10.1093/nar/gkp972 19906699 PMC2808936

